# Tuberculosis Epidemiology and the Impact of the COVID-19 Pandemic on Tuberculosis Control in Tétouan, Morocco (2015–2022): A Retrospective Descriptive and Analytical Study

**DOI:** 10.7759/cureus.81467

**Published:** 2025-03-30

**Authors:** Ayoub Ez-Zari, Laila Farouk, Nadya Mezzoug, Zakaria Mennane, Khalid Bouti, Noureddine El Mtili

**Affiliations:** 1 Department of Biology, Laboratory of Biology and Health (UAE/U06FS), Abdelmalek Essaâdi University, Faculty of Science, Tetouan, MAR; 2 Pulmonology Department, Mohammed VI University Hospital, Tangier, MAR

**Keywords:** anti-tuberculosis, lost to follow up, sars-cov2 pandemic, surveillance, treatment success, tuberculosis

## Abstract

Introduction: Tuberculosis (TB) is a major public health problem in Morocco, specifically in the Tangier-Tetouan-Al-Hoceima region, which includes the city of Tetouan. Although the improvement in the epidemiological situation in Tetouan over the past two decades, this remains insufficient to achieve the ultimate goal of eradicating tuberculosis. The present study describes and analyzes tuberculosis's epidemiological status in Tetouan between 2015 and 2022 to understand the evolution of infection, especially before and during the COVID-19 pandemic.

Methods: A retrospective study was conducted on tuberculosis patients registered in Tetouan from January 2015 to December 2022. Data on demographic characteristics and clinical tuberculosis presentations were analyzed using IBM SPSS Statistics software (IBM Corp., Armonk, NY, USA), with significance determined at a p-value < 0.05.

Results: A total of 6,397 TB cases were reported between 2015 and 2022, with an average incidence rate of 127.33 per 100,000 inhabitants. Males were more frequently affected, with a sex ratio of 1.64. The majority of cases occurred in young adults (15-34 years), with an average patient age of 39.69 years. New cases accounted for 5,751 (89.90%) of the total. Intra-pulmonary tuberculosis was the most common form, representing 3,612 cases (56.50%), of which 3,038 (88.39%) were bacteriologically confirmed. Among extra-pulmonary tuberculosis cases, pleural tuberculosis was the most prevalent, affecting 1,220 patients (43.79%). The cure rate was 5,236 (89.87%), while mortality remained below 3% (159 cases). The treatment dropout rate was 4.28% (249 cases). The COVID-19 pandemic led to a significant decline in TB notifications and an increase in the number of patients lost to follow-up.

Conclusion: Despite the favorable epidemiological evolution of the disease in Tetouan, many efforts are essential for more case detection and to overcome the impact of the COVID-19 pandemic.

## Introduction

Tuberculosis (TB) remains a major global health threat, ranking as the second deadliest infectious disease after COVID-19 and surpassing HIV in mortality [1]. Primarily affecting young adult men, over 10.6 million new tuberculosis cases were reported globally in 2021, with 1.6 million deaths [2]. Between 2015 and 2020, TB incidence declined by 2% annually, totaling an 11% reduction, although the World Health Organisation (WHO) had projected a 20% decrease for the same period [3].

In Morocco, the incidence rate of tuberculosis in 2019 was 97 cases per 100,000 inhabitants, with a variable distribution across regions [4]. The highest incidence rate was observed in the northwestern Mediterranean region (including the three cities of Tangier, Tetouan and El-Hoceima), which reported 115 cases per 100,000 inhabitants in 2019 [5]. Despite a decline from 213 cases per 100,000 in 2005 to 142 in 2019, Tetouan’s incidence remains above the national average [4].

Given that existing tuberculosis control strategies remain insufficient [1], the COVID-19 pandemic has further exacerbated the situation [2], hindering progress in reducing the global TB burden [6]. Therefore, this study aims to achieve multiple objectives: (1) provide a descriptive analysis of TB epidemiology in Tetouan from 2015 to 2022, (2) assess the impact of the COVID-19 pandemic on TB control services, (3) analyze trends in TB case notification and treatment outcomes, and (4) compare TB data between the pre-pandemic (2015-2019) and pandemic (2020-2022) periods.

In this study, WHO definitions [1] are used, reflecting Morocco’s strict adherence to the WHO’s treatment, diagnosis and follow-up guidelines. A tuberculosis case is any person with typical TB symptoms and/or radiological signs [1] who starts anti-TB treatment regardless of bacteriological confirmation. Pulmonary TB with bacteriological confirmation (PTB+) when sputum tests positive via GeneXpert MTB/RIF Ultra (Cepheid Inc, Sunnyvale, CA, USA) or smear microscopy, while clinically confirmed pulmonary TB (PTB-) is diagnosed based on clinical and radiographic findings despite negative bacteriology. Extrapulmonary TB (EPTB) is defined as tuberculosis affecting organs other than the lungs and is diagnosed through clinical, radiological, histopathological, or bacteriological evidence. PTB and EPTB represent TB forms classified by localization. Primary tuberculosis infection (PTI) is the initial infection with Mycobacterium tuberculosis, often asymptomatic or with non-specific symptoms. It is diagnosed through immunological tests, such as the tuberculin skin test (TST) and interferon-gamma release assays (IGRAs).

New TB cases include patients never treated or those who discontinued treatment for less than one month. Retreatment covers both relapse (TB recurrence after treatment completion) and treatment failure/lost to follow-up (patients restarting therapy due to inadequate response or prolonged treatment interruption). A patient is considered cured if bacteriological tests become negative after treatment, whereas treatment completed refers to patients showing clinical improvement without bacteriological confirmation. Treatment failure occurs when PTB+ remains positive after five months, and death refers to TB-related fatalities during or before treatment. Lost to follow-up is defined as patients who either never initiate treatment after diagnosis or who discontinue their treatment for two or more consecutive months during the treatment period, and therapeutic success includes both cured and treatment-completed cases.

## Materials and methods

Type and study setting

This is a retrospective, descriptive, and analytical study of tuberculosis patients registered and followed up at the Tuberculosis and Respiratory Diseases Diagnostic Center (CDTMR) in Tetouan, Morocco, over an eight-year period (from January 1, 2015, to December 31, 2022). Tetouan is a city in the Tangier-Tetouan-Al Hoceima region, located in the northwest of the country, with a population of over 563,000 in 2020.

Data collection methods and instruments

Data were extracted from annual registers and clinical records of patients diagnosed and monitored at the city’s TB center. The collected clinical and demographic information was systematically organized into annually updated tables. To ensure data accuracy and reliability, verification procedures were applied, including cross-checking records and quality control measures by more than two Investigators. Population denominators were derived from official health reports and census data.

Inclusion criteria

Data on tuberculosis patients registered during the study period were systematically collected and analyzed. All registers were well-maintained, with no missing or damaged records. Additionally, electronic data records were utilized to enhance data integrity.

Data analysis

Data were categorized based on demographic characteristics (age groups, mean age, sex) and clinical status related to TB (form, type, history, localization and treatment outcome). The study period was divided into two timeframes, before (2015-2019) and during (2020-2022) the COVID-19 pandemic, to assess the impact of the pandemic on tuberculosis statistical variables. Descriptive statistics (percentages, standard deviations, prevalence), regression (binomial and multinomial), comparison tests (Student's T-test) and correlation tests ( (r) Pearson correlation) were conducted using IBM SPSS Statistics v26 software (IBM Corp., Armonk, NY, USA), with a significance threshold set at a P-value of less than 0.05.

Ethical considerations 

All data were treated anonymously and used only for the purposes assigned to the study. The study protocol was approved by the Institutional Review Board (IRB) of the Hospital-University Ethics Committee of Tangier (CEHUT) (IRB No: AC112JV/2-025). The informed consent waiver was granted by the IRB since our retrospective study exclusively used de-identified patient records, thereby eliminating the need for direct patient interaction.

## Results

Annual incidence rate

The annual case notification average of TB between 2015 and 2022 is 785 (±75), with a total of 6397 TB cases (all TB-forms included).

From 2005 to 2022, the annual incidence of tuberculosis in Tetouan has exhibited considerable fluctuations, decreasing significantly from 213 per 100,000 inhabitants in 2005 to 150 per 100,000 in 2008. Subsequently, it rose to 201 per 100,000 inhabitants in 2013 before dropping again to 133 per 100,000 inhabitants in 2017. The incidence then slightly increased to 142 per 100,000 inhabitants in 2019, followed by a sharp decline to 112 per 100,000 inhabitants in 2020. After this drop, the incidence of TB cases remained stable for the next two years, 2021 and 2022.

**Figure 1:**
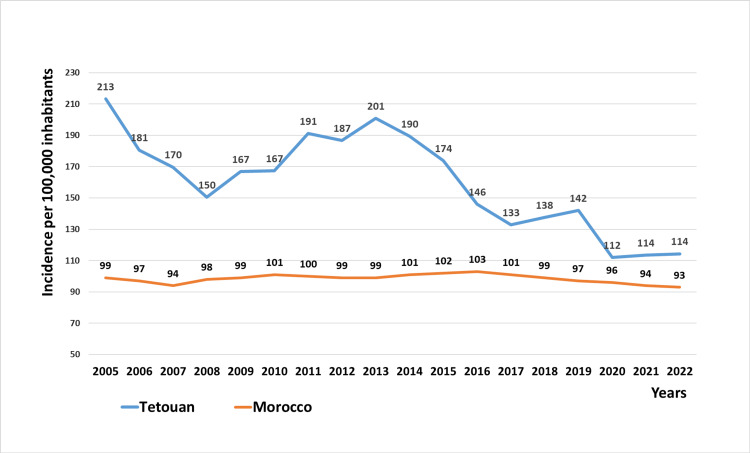
Tuberculosis incidence trends in Tetouan and Morocco (2005-2022)

These fluctuations contrast sharply with the national trends, where TB case rates have been much more stable, varying between 103 in 2017 and 93 in 2022 (Figure 1).

Distribution of TB cases by age

Between 2015 and 2022, tuberculosis affected individuals across all age groups, with the highest proportion of cases occurring in the 15-34 age group (n=2,863, 44.76%). Children under 15 years accounted for 4.17% (n=267) of cases, while pediatric forms under five years were rare (n=38, 0.98%). Patients aged 65 years and older represented 9.48% (n=692) of the total cases (Table 1).

**Table 1:** Tuberculosis epidemiological profile between 2015 and 2022 in Tetouan, Morocco (N=6,397) n*: Number of cases with known outcome; n**: Total of cases tested for drug resistance; TB: Tuberculosis; RR: Rifampicin resistance; MDR: Multi-drug resistance (resistance to rifampicin and isoniazid); Polyresistance: Resistance to rifampicin and other second-line anti-tuberculosis drugs.

Variables	n	(%)
Gender		
Male	3974	62.12
Female	2422	37.87
TB status		
New cases	5751	89.9
Retreatment (after relapse)	484	7.56
Retreatment (treatment failure/lost to follow-up)	16	2.53
Age group (years)		
< 5	38	0.59
5-14	229	3.58
15-24	1414	22.10
25-34	1449	22.66
35-44	1043	16.31
45-54	810	12.66
55-64	720	11.25
≥ 65	692	10.84
Tuberculosis form		
Pulmonary TB	3612	56.5
Extra-pulmonary TB	2701	42.3
Primary tuberculosis infection	75	1.17
Pulmonary TB diagnosis		
Bacteriologically confirmed	3038	84.1
Clinically confirmed only	573	15.9
Treatment outcome (n*=5,826)		
Therapeutic success	5236	89.87
Treatment failure	46	0.79
Deaths	159	2.75
Transfer/other	134	2.31
Lost to follow-up	249	4.28
RR/MDR tuberculosis (n**=5,177)		
RR	8	0.12
MDR	6	0.11
Polyresistance	2	Rare
All resistance cases	16	0.32

**Figure 2:**
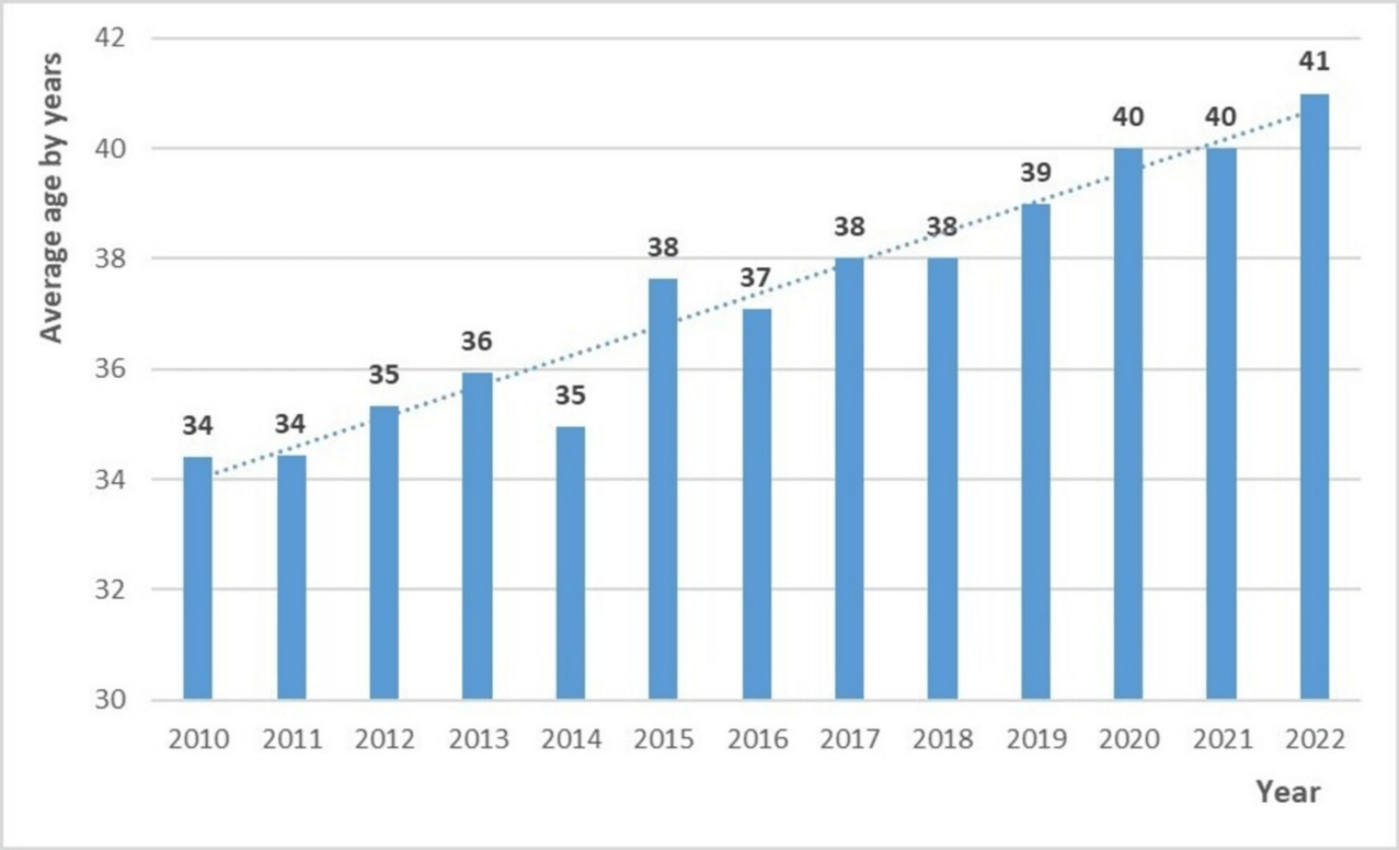
Average age fluctuations among tuberculosis patients registered in Tetouan, Morocco, between 2010 and 2022

The average age of all cases between 2015 and 2022 was 39.69 (±19.01) years, while the average age calculated between 2010 and 2014 was 35 (±24.32) years. Overall, the average age showed a linear increase from 34 years in 2010 to 41 years in 2022 (p=0.002). The disease’s epidemiological evolution indicates a deviation towards older ages (Figure 2).

Distribution of TB cases by gender (2015-2022)

**Figure 3:**
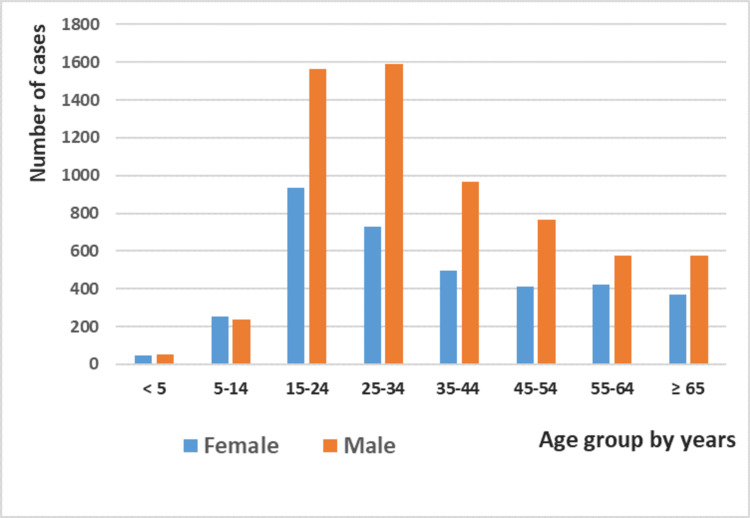
Distribution of tuberculosis cases by age group and gender in Tetouan, Morocco, from 2015 to 2022 (N=6,397)

Men were more affected by tuberculosis (all forms combined) than women (sex ratio = 1.64), a trend that remained consistent throughout the study period (Table 1). This predominance was observed across all age groups from 15 years to over 65. However, in pediatric cases (under 15 years), tuberculosis affected both genders equally (r=0.888, p<0.001) (Figure 3).

Distribution of TB cases by form (2015-2022)

The overall incidence of PTB was 56.53% (n=3,612), while EPTB accounted for 42.30% (n=2,701), and 75 patients (1.17%) were diagnosed with PTI. Over the eight-year study period, the distribution of TB cases remained consistent, with PTB cases consistently outnumbering EPTB cases and primary TB infection remaining below 2%. Bacteriologically confirmed PTB+ cases represented the majority of PTB cases, averaging 84.1% annually (Table 1).

**Figure 4:**
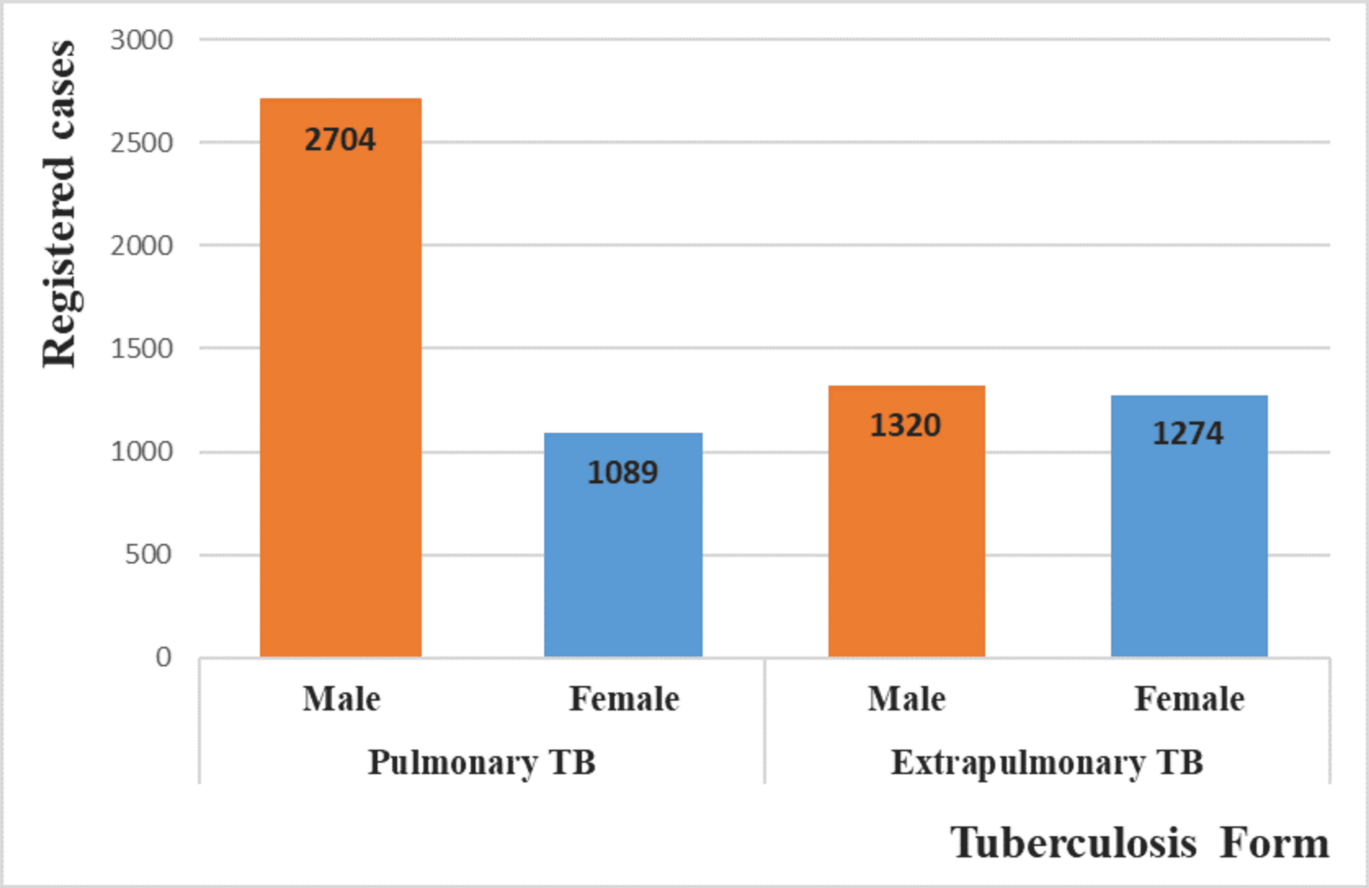
Distribution of reported tuberculosis cases by gender and tuberculosis (TB) form in Tetouan, Morocco, from 2015 to 2022 (N=6,397)

Women faced a significantly increased risk of developing EPTB compared to men, with a relative risk 1.67 times higher (95% CI = [1.37-2.03], p <0.001). In contrast, PTB cases were more prevalent among men, with a relative risk of 2.23 times higher (95% CI = [1.647-3.033], p<0.001) (Figure 4).

**Figure 5:**
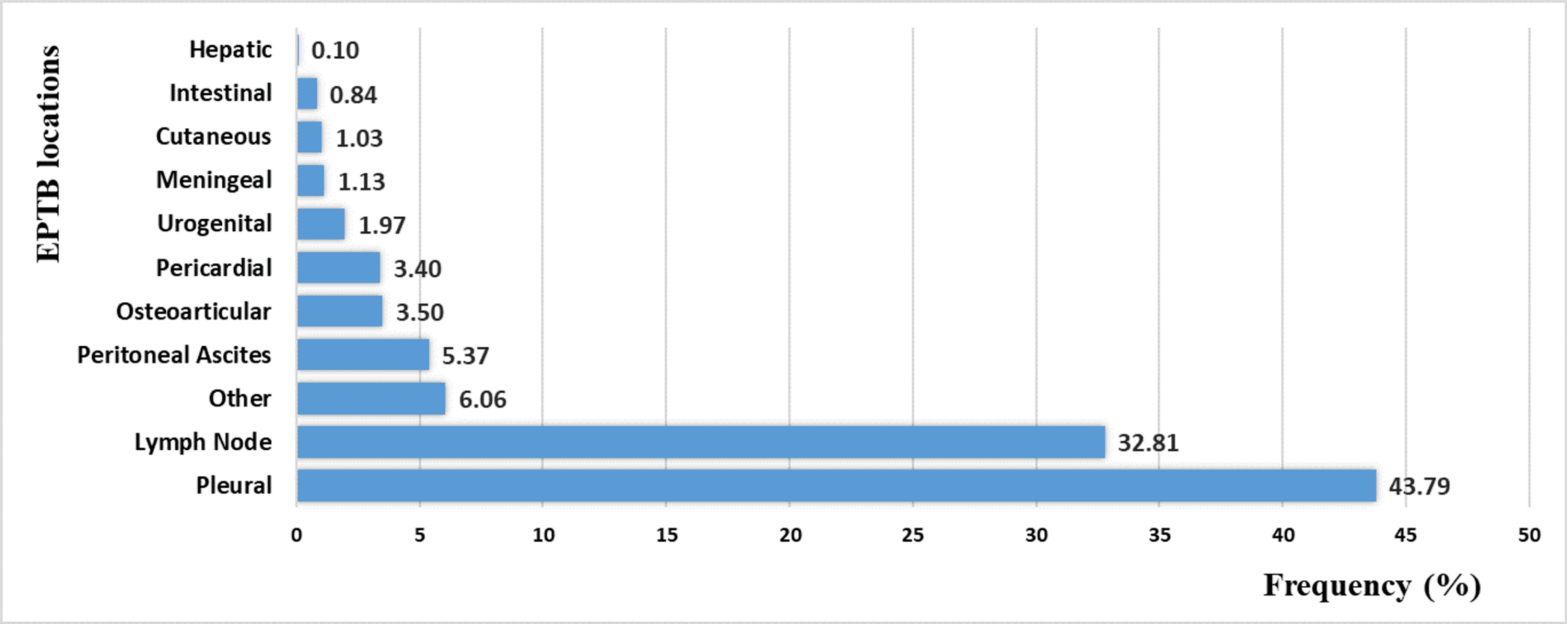
The distribution of extra-pulmonary tuberculosis (EPTB) cases by anatomical localization in the city of Tetouan, Morocco (2015-2022)

The most common location affected by EPTB is the pleural cavity (n=1,183, 43.79%), followed by the lymph nodes (n=886, 32.8%). The liver is the rarest affected location with only three cases (0.1%), and tuberculous meningitis accounts for 30 cases (1.13%) (Figure 5).

Distribution of TB cases by TB patient’s history (TB status) (2015-2022)

Out of the 6,397 cases, the highest number of new cases was recorded in 2015, with 865 cases. In 2020, this number declined to 595 cases, and then showed a moderate increase in 2021 and 2022 to 589 and 604 cases, respectively. During the study period, there has been no statistically significant change in the new case rate, with an overall average of 89.9% of all registered cases. Otherwise, there has been a noticeable decline in the rate of re-treatment cases after relapse, decreasing from 8.45% in 2015 to 5.91% in 2022 (p=0.038), with an annual average of 7.56% (Table 1).

Outcome of tuberculosis patients (2015-2022)

For cases with known outcomes (N=5,826), the therapeutic success rate was 89.87% (n=5,236), exceeding 87% annually. A total of 159 patients (2.75%) died, 249 (4.28%) were lost to follow-up, and the treatment failure rate was 0.79% (46 patients) (Table 1).

Impact of COVID-19 on TB epidemiology

To evaluate the impact of the COVID-19 pandemic on tuberculosis indicators, the study period was divided into two parts: from 2015 to 2019, considered before the emergence of COVID-19, and from 2020 to 2022, when COVID-19 was officially declared a global pandemic by the WHO.

The incidence of tuberculosis showed a significant decrease of 18.74% between the two periods, dropping from 140.33 to 114.33 per 100,000 inhabitants (p=0.019). There were no statistically significant variations observed before and after the COVID-19 pandemic in the distribution of cases by gender and TB status.

For the distribution by age group, no difference was observed between the two periods except for pediatric cases under five years old, which decreased from 0.65% to 0.36% (p=0.037). The mean age of TB cases continued to rise, reaching 41 years in 2022. The distribution of TB cases by type did not change significantly between the two periods. However, there was a notable increase in the rate of bacteriologically confirmed pulmonary TB cases (p=0.010), alongside a decrease in bacteriologically negative pulmonary TB cases (p=0.009).

The treatment outcomes remained largely the same between the two periods, except for a statistically significant rise in the rate of lost to follow-up cases, rising from 3.61% before COVID-19 to 7.66% during the pandemic (p=0.023) (Table 2).

**Table 2:** Tuberculosis descriptive statistics patients in Tetouan, categorized by periods (before the COVID-19 pandemic: 2015–2019 vs. during the pandemic: 2020–2022), N=6,397 Av: Annual average; PTB: Pulmonary tuberculosis; PTB (-): Clinically confirmed; PTB (+): Bacteriologically confirmed; EPTB: Extra-pulmonary tuberculosis; PTI: Primary tuberculosis infection; *: p < 0.05 means the two averages are significantly different (CI=95%); n**: number of patients included in the variable’s study

Variable	Pre-COVID-19 (2015-2019)	During COVID-19 (2020-2022)	P value*
Mean number of cases/year (± SD)	799 (96.55)	653 (6.24)	-----
Incidence (/100,000 inhab.) (± SD)	140 (19.92)	114 (0.57)	0.019
Distribution of cases by gender Av (%)			
Male	517 (63.26)	392 (60.98)	0.095
Female	300 (36.73)	251 (39.01)	0.095
Distribution of cases by TB status Av (%)			
New cases	758 (89.62)	596 (90.19)	0.511
Retreatment after relapse	67 (7.87)	48 (7.25)	0.162
Retreatment (Failed treatment and lost to follow-up)	21 (2.50)	17 (2.56)	0.610
Distribution of cases by age group Av (%)			
< 5	5 (0.65)	2 (0.36)	0.037
5-14	31 (3.65)	20 (2.64	0.244
15-24	191 (22.48)	125 (19.07	0,300
25-34	192 (22.67)	142 (22.75)	0.936
35-44	129 (15.34)	135 (18.71)	0.08
45-54	104 (12.37)	91 (13.72)	0.313
55-64	96 (11.43)	73 (11.30)	0.903
≥ 65	96 (11.40)	60 (9.44)	0.083
Average age (years)	38.48	40.90	0.018
Distribution of TB cases by form Av (%)			
PTB	472 (57.27)	354 (55.74)	0,418
EPTB	342 (41.40)	320 (44.05)	0.275
PTI	11 (1.31)	3 (0.20)	0.054
Pulmonary TB diagnosis			
PTB (+)	394 (83.54)	312 (93.25)	0.010
PTB (-)	78 (16.54)	43 (6.74)	0.009
Treatment outcome (n**=5,826) Av (%)			
Therapeutic success	745 (90.46)	577 (88.97)	0.339
Treatment failure	6 (0.68)	6 (0.98)	0.832
Deaths	23 (2.85)	17 (2.62)	0.891
Transfer	21 (2.39)	11 (1.78)	0.169
Lost to follow-up	24 (3.61)	37 (7.66)	0.023

Determinant factors of lost to follow-up cases (2015-2022)

Although age does not have a significant impact on treatment abandonment rates, factors such as gender, TB form and status are statistically linked to treatment discontinuation (Table 3).

**Table 3:** Determinants of tuberculosis patients lost to follow-up in Tetouan from 2015 to 2022 (N=5,826) ORa: Adjusted odds ratio; *: p < 0.05 means the two averages are significantly different (CI=95%); PTB: Pulmonary tuberculosis; EPTB: Extra pulmonary tuberculosis; Relapses#: Including cases lost to follow-up and treatment failure.

Determinant	Total number of TB patients, n (%)	Lost to follow-up		OR^a^ (95% CI)	P value
		YES n (%)	NO n (%)		
Gender					
Male	3898 (66.9)	340 (86.7)	3558 (34.5)	3.424 (1.422-8.21)	0.006*
Female	1927 (33.1)	52 (13.3)	1875 (65.5)	1	
Age (years)					0.251
< 24	1395 (24)	61 (15.6)	1333 (24.6)	0.732(0.285-1.878)	
25-34	1483 (25.4)	112 (28.9)	1368 (25.2)	1.325(0.598-2.937)	
35-44	1019 (17.5)	105 (26.7)	916 (16.9)	1.829(0.806-4.147)	
≥ 45	1927 (33.1)	112 (28.9)	1814 (33.4)	1	
TB Status					0.003*
Relapse^#^	384(6.6)	70(17.8)	314(5.8)	3.526(1.530-8.126)	
New cases	5441(93,4)	323 (82.2)	5119 (94.2)	1	
TB form					0.011*
PTB	3454 (59.3)	304(77.8)	3149(57.9)	2.538(1.236-5.208)	
EPTB	2371 (40.7)	87 (22.2)	2285(42.1)	1	

## Discussion

Reducing TB incidence by at least 20% between 2015 and 2020 and reaching a 50% reduction by 2025 are key targets within the WHO's Sustainable Development Goals (SDGs) and the End-TB strategy [7]. In Tetouan, a reduction of 18.4% was registered between 2015 and 2020, which is slightly below the WHO’s milestone. During the study period, TB incidence fluctuated three times. First, between 2015 and 2017, the incidence decreased markedly, continuing the downward trend that began in 2013, which aligned with the launch of the national plan "2013-2016 TB Strategy", aimed at accelerating TB reduction, especially in high-prevalence cities [8]. Subsequently, the TB notifications increased again, peaking in 2019. This rise can be attributed to the introduction of molecular testing (GeneXpert® MTB/RIF Ultra). In 2020, the prevalence dropped significantly due to the negative impact of the COVID-19 pandemic on the national TB control program. Afterward, the incidence remained stable over the next two years.

Despite the overall decline in TB notifications, Tetouan maintained a significantly higher disease burden compared to nationwide data. In 2019, Tetouan’s case notification rate was 31.7% higher than the national average [4]. Several factors can explain this difference, including inadequate ventilation and overcrowding in several city districts, which can facilitate pathogen spread [9]. Tetouan is also a major transit point for illegal immigrants from high-TB-burden African countries seeking to cross into Europe [10].

Our findings reveal that tuberculosis affects all age groups, with individuals aged 15 to 34 years being the most affected (44.76%), indicating that TB primarily targets the younger, active population in Tetouan. These results are consistent with both national [4,11-14] and international data [15-17]. Therefore, targeted screening and awareness campaigns for the working-age population are essential. Health promotion activities - such as educational initiatives on TB symptoms, treatment management, and transmission risk factors, alongside workplace interventions and community engagement programs - play a crucial role in TB control. These efforts facilitate early detection, enhance treatment adherence, and help reduce TB transmission, particularly in workplace and community settings.

Active tuberculosis remains rare in children under 15 years old (4.17% of all cases). Over the past decade, pediatric TB cases have decreased from 7.8% in 2010 to 5.3% and 3% in 2015 and 2020 respectively. Our results align with those found in several Moroccan cities, such as Settat [12], Laayoune [13], and Marrakech [11], as well as nationwide [4]. This trend could be linked to the vaccination policy adopted at the national level, advocating for compulsory bacille Calmette-Guerin (BCG) vaccination at birth [18], which has been validated as an effective measure in reducing pediatric TB transmission [19].

Patients over 65 years old represent 10.84% of all cases, which is consistent with nationwide results [4]. Internationally, the rate of elderly TB cases varies significantly, ranging from 8.3% to 34%, and tends to rise as the overall incidence declines [11-13,16,17,20,21]. Although a sedentary lifestyle reduces their exposure, their vulnerability raises the risk of active TB due to new infections or latent reactivation [15]. Additionally, the average patient age has risen from 34 in 2010 to 41 in 2022 (p=0.018) in Tetouan, mirroring national trends [4], suggesting an epidemiological shift toward older populations.

Men were more affected by TB (M/F sex ratio = 1.64), with this disparity observed across all age groups except for children under 14 years, where both genders were equally affected. This finding aligns with several national and international studies [4,11-13,20,22,23].

The rate of PTB (56.5%) was slightly higher than that of EPTB (42.3%), consistent with national studies [4,12-14]. Internationally, PTB rates range from 50.6% to 89%, while EPTB is more common in low-incidence countries [16,17,20,23-26]. In Tetouan, while PTB was primarily confirmed through bacteriological methods (GeneXpert and smear microscopy) (84.1%), the remaining clinically diagnosed cases (based on clinical symptoms and/or radiography) often pose a considerable risk of misdiagnosis, jeopardizing the patient’s prognosis [27]. For EPTB, diagnosis relies mainly on biopsy and histopathology or biochemical tests (such as adenosine-deaminase dosage (ADA) for pleural TB), which can lead to either underestimation or overestimation of case numbers [28].

A significant association was found between gender and TB form, with men more prone to PTB and women to EPTB, aligning with national [11-13] and international [24,26,29,30] data. While men’s higher PTB risk stems from greater exposure to airborne infections due to travel, social interactions, and smoking [31], EPTB is associated with risk factors beyond airborne transmission, including comorbidities such as diabetes, extreme age (under 15 or beyond 60 years) and female gender [32]. The exact reasons for women’s increased susceptibility to EPTB remain poorly understood. However, genetic and hormonal factors are potential contributors [33], along with behavioral factors such as smoking [26,32]. Given that smoking is less common among women in Morocco [34], it may contribute to their lower PTB incidence and higher EPTB prevalence in Tetouan.

EPTB localizations were polymorphic, with pleural TB being most common (43.79%), followed by lymph node TB (32.81%). These two forms are also the most frequently reported in several studies; however, some report pleural TB as the predominant type [14,24,25,29] while others cite lymph node TB [11-13,17,20].

Tuberculous meningoencephalitis typically represents 1-2% of all EPTB cases in high-burden regions and can be fatal or lead to severe neurological complications [35]. During the study period, 23 patients (1.13% of EPTB cases) developed tuberculous meningoencephalitis, a proportion slightly higher than that found in Settat, Morocco (0.79%) [13], but lower than in other studies, which report rates ranging from 1.6% to 11.68% [13,16,20,26,29]. Severe EPTB forms, like tuberculous meningitis and miliary TB, are linked to immune status, HIV, and vaccination. Morocco’s mandatory BCG vaccine and low HIV/TB co-infection rate [4] may explain Tetouan’s low meningoencephalic TB incidence, though underdiagnosis or delayed diagnosis remain possible [28].

Among the 6,397 cases recorded (2015-2022), 89.9% were new, and 7.56% were relapses. The relapse rate is slightly higher than in Laayoune, Morocco (6.6%) [14], Sabah, Malaysia (6%) [21] and Canada (5.3%) [16]; but lower than in Recife, Brazil (28.1%) [23]. Several factors can increase the risk of relapse, including treatment dropout, malnutrition, comorbidities (e.g., HIV), and living in high-TB-prevalence settings [36].

Relapse cases may result from either strain reactivation or reinfection, but molecular analysis [37] is needed to distinguish between these two possibilities in Tetouan. Stable low retreatment rates (2015-2022) suggest treatment effectiveness and explain the low drug-resistant TB incidence (16 cases, 0.32%).

Therapeutic success rate exceeded 89%, surpassing the national average (85%) [4]. Tetouan's regimen remained largely unchanged from 2015 to 2022, with individualized treatment and continuous monitoring ensuring higher cure rates than the WHO's 2018-2019 figures (85-86%) [1]. Additionally, TB-related mortality was 2.75%, similar to Settat (2.76%) [12] and well below the national average fatality rate (10%) [4,8], as well as the 8% rate reported in Sabah, Malaysia [21], highlighting the effectiveness of Tetouan’s treatment strategy.

Patients lost to follow-up accounted for 7.06%, which was lower than that registered in Settat, Morocco (15.5%) [13], and higher than Sabah, Malaysia (2%) [21] and Canada (0.1%) [16]. Treatment dropout poses a significant challenge to TB eradication efforts in Tetouan. Several determinants have been linked to non-compliance with treatment. Firstly, men were more than three times as likely to drop out of treatment compared to women (3.424 [1.422-8.21], p=0.006), which is consistent with findings from other studies [14,36]. Moreover, pulmonary TB patients faced a higher risk of treatment discontinuation than those with extrapulmonary TB (2.538 [1.236-5.208], p=0.011), consistent with findings from Valencia (Spain) [32] and Settat (Morocco) [12]. Furthermore, retreatment cases were also more likely to abandon treatment than new cases (3.526 [1.530-8.126], p=0.003), highlighting a recurrence of non-adherence.

Comparing the two periods before and during the COVID-19 pandemic, we noted a significant decline of 18.74% in TB case notifications, reflecting the worldwide trend, with an alarming global drop of up to 42% in TB case detection, especially in high-TB-burden countries [38]. In Tetouan, this sharp decrease in reported TB cases could be explained by several factors. One possibility is that fewer cases were detected due to reduced case identification; as COVID-19 spread, fear of the virus discouraged individuals from seeking care at the TB treatment and diagnostic center. Additionally, lockdown measures with a restriction on public transport facilities severely limited access to the TB diagnostic center. On the other hand, measures such as physical distancing and increased use of facemasks may have helped reduce TB transmission, as these strategies were found to lower TB transmission rates during COVID-19 by approximately 10% in countries with high TB burdens [39].

During the COVID-19 period, the number of bacteriologically confirmed pulmonary TB cases increased by 10% (p=0.010), while clinically diagnosed cases decreased by the same percentage (p=0.009). This trend might be due to the restricted access to the TB diagnostic center during lockdowns, when only severe cases - more likely to test positive for TB - were brought in for diagnostic assistance. Additionally, since TB PCR machines (GeneXperts MTB/Rif ultra) were not repurposed for COVID-19 detection, the biological screening rate was maintained.

Therapeutic success, mortality, and failure rates remained stable across both periods, likely due to consistent treatment management by healthcare centers throughout the city, ensuring that patients consistently received their medication. While this aligns with the 2022 WHO TB report [1], some high-burden regions reported poorer TB treatment outcomes during the pandemic due to lockdown measures, fear of contracting COVID-19 in hospitals, and the diversion of resources from routine anti-TB programs to focus on anti-COVID-19 protocols [40].

Despite stable treatment success, lost-to-follow-up cases rose by 40.5% (p=0.023) during the pandemic. Given the persistent issues with treatment dropouts in Tetouan’s anti-TB program, it was expected that the pandemic would worsen the situation. To address patients’ poor treatment compliance, two non-profit associations had been tasked with contacting patients who had discontinued their treatment, encouraging them to return to the TB center for check-ups and resume therapy. Additionally, tools such as mobile technology, Medication Event Monitoring Systems, and smart pillboxes were used to support adherence, alongside training health professionals to educate patients on the critical importance of treatment [41]. Unfortunately, these efforts were halted during the COVID-19 pandemic, resulting in a significant rise in lost-to-follow-up cases.

The present study has several limitations. First, its retrospective and cross-sectional design limits the ability to definitively infer causality, and potential surveillance bias may have been introduced due to pandemic-related disruptions in TB case reporting. Moreover, the unique characteristics of the studied region may restrict the generalizability of these findings to other settings. Additionally, the co-infection of HIV and TB was not comprehensively addressed since not all patients were tested for HIV, and key variables such as socio-economic factors and comorbidities were absent from the analysis. Finally, the lack of monitoring for treatment side effects and symptom evolution, as well as imprecise reporting of patients' addresses, further constrains our ability to accurately assess patient outcomes and regional TB distribution.

## Conclusions

In conclusion, while TB incidence in Tetouan is gradually declining, it remains a significant concern, with rates still exceeding the national average. Strengthening TB control requires several key measures. First, expanding access to screening by establishing testing sites across the city and ensuring free availability to the population. Additionally, investing in more sensitive and accurate diagnostic tools - such as the QuantiFERON-Gamma Release Assay and ADA testing for extrapulmonary TB - is crucial, as these tests remain costly. Second, targeted screening campaigns should focus on high-risk groups, including working-age men and immigrants from high-burden regions. Third, addressing treatment dropout is essential and requires enhanced patient education and follow-up through a multisectoral approach involving non-profit organizations. Finally, mitigating the long-term impact of the COVID-19 pandemic on TB programs necessitates reinforcing healthcare infrastructure by recruiting additional professionals and implementing robust protocols to optimize TB case management.

## References

[1] (2021). Global tuberculosis report. https://books.google.co.ma/books?hl=fr&lr=&id=DHkOEQAAQBAJ&oi=fnd&pg=PA1&dq=World+Health+Organization.+Global+tuberculosis+report+2021.+2021.+Geneva.&ots=lf5kjFiHra&sig=-GxBDv4SdOJuqBMACfKvgMPDsXU&redir_esc=y#v=onepage&q=World%20Health%20Organization.%20Global%20tuberculosis%20report%202021.%202021.%20Geneva.&f=false.

[2] Bagcchi S (2023). WHO’s global tuberculosis report 2022. Lancet Microbe.

[3] Raviglione M, Sulis G (2016). Tuberculosis 2015: burden, challenges and strategy for control and elimination. Infect Dis Rep.

[4] La Direction de l’Epidémiologie et de Lutte contre les Maladies M de la santé, R du Maroc. BULLETIN D’ÉPIDÉMIOLOGIE ET DE SANTÉ PUBLIQUE. 78th ed (2021). La Direction de l’Epidémiologie et de Lutte contre les Maladies M de la santé, R du Maroc. https://www.sante.gov.ma/Publications/Bullten_pidmiologique/BESP%2078%20DELM.pdf.

[5] Sadeq M, Bourkadi JE (2018). Spatiotemporal distribution and predictors of tuberculosis incidence in Morocco. Infect Dis Poverty.

[6] Falzon D, Zignol M, Bastard M, Floyd K, Kasaeva T (2023). The impact of the COVID-19 pandemic on the global tuberculosis epidemic. Front Immunol.

[7] Lönnroth K, Raviglione M (2016). The WHO's new End TB Strategy in the post-2015 era of the Sustainable Development Goals. Trans R Soc Trop Med Hyg.

[8] (2024). National strategic plan for tuberculosis prevention and control, Morocco 2024-2030. https://www.sante.gov.ma/Documents/2023/11/Plan%20strate%C3%ACgique%20National%20TB%202024-2030.pdf.

[9] Lienhardt C (2001). From exposure to disease: the role of environmental factors in susceptibility to and development of tuberculosis. Epidemiol Rev.

[10] Lahlou M (2018). Migration dynamics in play in Morocco: trafficking and political relationships and their implications at the regional level. MENARA.

[11] Bahi YE, Loukid M, RKha S (2024). Characteristics of tuberculosis in Marrakech (Morocco): epidemiology and related factors. Clin Epidemiol Glob Health.

[12] Chahboune M, Barkaoui M, Iderdar Y (2022). [Epidemiological profile and diagnostic and evolutionary features of TB patients at the Diagnostic Centre for Tuberculosis and Respiratory Diseases in Settat, Morocco]. Pan Afr Med J.

[13] Eddabra R, Neffa M (2020). Epidemiological profile among pulmonary and extrapulmonary tuberculosis patients in Laayoune, Morocco. Pan Afr Med J.

[14] Sbayi A, Arfaoui A, Janah H, Koraichi SE, Quyou A (2020). Epidemiological characteristics and some risk factors of extrapulmonary tuberculosis in Larache, Morocco. Pan Afr Med J.

[15] Caraux-Paz P, Diamantis S, de Wazières B, Gallien S (2021). Tuberculosis in the elderly. J Clin Med.

[16] Mounchili A, Perera R, Lee RS, Njoo H, Brooks J (2022). Chapter 1: Epidemiology of tuberculosis in Canada. Can J Respir Crit Care Sleep Med.

[17] Pedraz T, Herrera L, Vazquez MC, Ramírez-Rubio O, Cano R, Herrador Z (2024). The epidemiological situation of tuberculosis in Spain according to surveillance and hospitalization data, 2012-2020. PLoS One.

[18] (2023). Practical manual on vaccination for health professionals. https://www.sante.gov.ma/Publications/Guides-Manuels/Documents/manuel%20pratique%20sur%20la%20vaccination%202015%20.compressed.pdf.

[19] Roy A, Eisenhut M, Harris RJ (2014). Effect of BCG vaccination against Mycobacterium tuberculosis infection in children: systematic review and meta-analysis. BMJ.

[20] Goroh MM, Rajahram GS, Avoi R, Van Den Boogaard CH, William T, Ralph AP, Lowbridge C (2020). Epidemiology of tuberculosis in Sabah, Malaysia, 2012-2018. Infect Dis Poverty.

[21] Pescarini JM, Simonsen V, Ferrazoli L, Rodrigues LC, Oliveira RS, Waldman EA, Houben R (2018). Migration and tuberculosis transmission in a middle-income country: a cross-sectional study in a central area of São Paulo, Brazil. BMC Med.

[22] Organization WH (2022). Global tuberculosis report 2021. https://iris.who.int/bitstream/handle/10665/363752/9789240061729-eng.pdf?sequence=1.

[23] de Lima Filho CA, de Oliveira IM, da Silva GE (2022). [Perfil epidemiológico da tuberculose em um município prioritário de Pernambuco no período de 2015-2020]. Res Soc Devel.

[24] Li T, Yan X, Du X (2023). Extrapulmonary tuberculosis in China: a national survey. Int J Infect Dis.

[25] Tahseen S, Khanzada FM, Baloch AQ (2020). Extrapulmonary tuberculosis in Pakistan- a nation-wide multicenter retrospective study. PLoS One.

[26] Sunnetcioglu A, Sunnetcioglu M, Binici I, Baran AI, Karahocagil MK, Saydan MR (2015). Comparative analysis of pulmonary and extrapulmonary tuberculosis of 411 cases. Ann Clin Microbiol Antimicrob.

[27] Siddiqi K, Lambert ML, Walley J (2003). Clinical diagnosis of smear-negative pulmonary tuberculosis in low-income countries: the current evidence. Lancet Infect Dis.

[28] Norbis L, Alagna R, Tortoli E, Codecasa LR, Migliori GB, Cirillo DM (2014). Challenges and perspectives in the diagnosis of extrapulmonary tuberculosis. Expert Rev Anti Infect Ther.

[29] Gomes T, Reis-Santos B, Bertolde A, Johnson JL, Riley LW, Maciel EL (2014). Epidemiology of extrapulmonary tuberculosis in Brazil: a hierarchical model. BMC Infect Dis.

[30] Rolo M, González-Blanco B, Reyes CA, Rosillo N, López-Roa P (2023). Epidemiology and factors associated with extra-pulmonary tuberculosis in a low-prevalence area. J Clin Tuberc Other Mycobact Dis.

[31] Rhines AS (2013). The role of sex differences in the prevalence and transmission of tuberculosis. Tuberculosis (Edinb).

[32] Arnedo-Pena A, Romeu-Garcia MA, Meseguer-Ferrer N (2019). Pulmonary versus extrapulmonary tuberculosis associated factors: a case-case study. Microbiol Insights.

[33] Ben Ayed H, Koubaa M, Marrakchi C (2018). Extrapulmonary tuberculosis: update on the epidemiology, risk factors and prevention strategies. Int J Trop Dis.

[34] Nejjari C, Benjelloun MC, Berraho M (2009). Prevalence and demographic factors of smoking in Morocco. Int J Public Health.

[35] Britz E, Perovic O, von Mollendorf C (2016). The epidemiology of meningitis among adults in a South African province with a high HIV prevalence, 2009-2012. PLoS One.

[36] Dooley KE, Lahlou O, Ghali I, Knudsen J, Elmessaoudi MD, Cherkaoui I, El Aouad R (2011). Risk factors for tuberculosis treatment failure, default, or relapse and outcomes of retreatment in Morocco. BMC Public Health.

[37] Jagielski T, van Ingen J, Rastogi N, Dziadek J, Mazur PK, Bielecki J (2014). Current methods in the molecular typing of Mycobacterium tuberculosis and other mycobacteria. Biomed Res Int.

[38] Ponnampalli S, Birudukota NV (2023). The impact of COVID-19 pandemic in high-burden countries for tuberculosis: a systematic review. Health Sci Rev.

[39] Quaife M, van Zandvoort K, Gimma A (2020). The impact of COVID-19 control measures on social contacts and transmission in Kenyan informal settlements. BMC Med.

[40] Caren GJ, Iskandar D, Pitaloka DA, Abdulah R, Suwantika AA (2022). COVID-19 pandemic disruption on the management of tuberculosis treatment in Indonesia. J Multidiscip Healthc.

[41] Park S, Moon N, Oh B, Park M, Kang K, Sentissi I, Bae SH (2021). Improving treatment adherence with integrated patient management for TB patients in Morocco. Int J Environ Res Public Health.

